# Computed tomography-derived body composition parameters as potential predictors of severity and adverse outcomes in viral pneumonia: a retrospective study

**DOI:** 10.7717/peerj.21619

**Published:** 2026-08-11

**Authors:** Xiujing An, Zhe Wu, Chao Jiang, Ning Li, Jubing Wan, Jinhua Wang, Yi Tang, Zheyong Li, Yang Cai, Lan Song, Lufeng Tian

**Affiliations:** 1 Postgraduate Training Base of Fushun Central Hospital of Jinzhou Medical University, Fushun, China; 2 Department of Internal Medicine, Fushun Central Hospital, Fushun, China; 3 Department of Radiology, Fushun Central Hospital, Fushun, China; 4 Department of Radiology, State Key Laboratory of Complex Severe and Rare Diseases, Peking Union Medical College Hospital, Chinese Academy of Medical Sciences and Peking Union Medical College, Bejing, China; 5 Department of Endocrinology, Fushun Central Hospital, Fushun, China; 6 Medical Records Office, Fushun Central Hospital, Fushun, China

**Keywords:** Viral Pneumonia, Tomography, X-ray Computed, Visceral fat, Sarcopenia, Disease severity, Body composition, Adverse outcomes, Clinical information, Prognosis

## Abstract

**Background:**

Following the COVID-19 pandemic, co-infections with multiple respiratory viruses have become increasingly common, complicating the accurate prediction of disease progression and prognosis. This study assessed the use of computed tomography (CT)-derived body composition parameters combined with clinical risk factors to predict the severity and short-term adverse outcomes of viral pneumonia.

**Methods:**

A total of 140 hospitalized patients with viral pneumonia who had undergone chest CT were retrospectively included and stratified into severe and non-severe groups. Body composition, including visceral and subcutaneous adipose volumes (VAV and SAV) and erector spinae volume (ESV), was measured at T4, T8, and T12 costovertebral joint levels and the corresponding whole-vertebral level using 3D-Slicer. Serological indicators and 30-day adverse outcomes were recorded. Clinical, imaging, and integrated models were developed to distinguish patients’ severity and outcome.

**Results:**

The median age was 77 years (IQR: 68–85), with 91 (65%) patients being male. Sixty-five (46.4%) patients were severe patients and 26 (18.6%) had adverse outcomes within 30 days. Lower T12-ESV and higher T12-VAV were associated with viral pneumonia severity after adjusting for confounders (*OR* = 0.823, 95% CI [0.733–0.924], *p* = 0.001; *OR* = 1.030, 95% CI [1.014–1.047], *p* < 0.001). Those with adverse outcomes had reduced subcutaneous fat (*p* < 0.05). The area under the curve (AUC) of the integrated model using clinical and body composition parameters was the highest (*AUC* = 0.843, *p* < 0.001).

**Conclusion:**

Decreased ESV and increased VAV at T12 level are independent predictors of severe viral pneumonia, while reduced SAV correlates well with adverse 30-day outcomes. CT-derived body composition parameters can predict the progression and short-term prognosis of viral pneumonia, thereby aiding clinical decision-making.

## Introduction

Respiratory viruses contribute to 20–40% of community-acquired pneumonia (CAP) cases in adults and are increasingly acknowledged as a leading cause of the disease (Cilloniz et al., 2024; Cillóniz et al., 2022). Viral pneumonia poses a significant burden on public health systems due to its high morbidity and mortality rates. Current evidence indicates that respiratory viruses are implicated in 15–45% of severe CAP cases (Nguyen et al., 2016; Karhu et al., 2014; Hasvold et al., 2016; Garg et al., 2015; Musher, 2020; Choi et al., 2012) and 20–40% of cases requiring intensive care unit (ICU) admission (Cilloniz et al., 2024; Cillóniz et al., 2022; Cilloniz et al., 2022). During the COVID-19 pandemic, the occurrence of over 700 million infections and approximately 6.5 million deaths underscored the critical need for effective severity and prognosis assessments in the management of viral pneumonia (World Health Organization, 2024). While stringent public health measures have curtailed the spread of other respiratory viruses, reduced exposure may have weakened host immunity, potentially heightening the risks of future epidemics (Cilloniz et al., 2024; Gomez, Mahé & Chaves, 2021). Furthermore, the heterogeneity of viral pneumonia makes it difficult to accurately predict the severity and outcomes of epidemics, underscoring the need for reliable clinical decision-making tools.

In recent years, quantitative computed tomography (QCT) has demonstrated distinct advantages in diagnosing and assessing pulmonary diseases (Kirby & Smith, 2023; Wu et al., 2024). Since the COVID-19 pandemic, CT-based quantitative imaging has gained widespread attention around the world, having been applied in diagnosing COVID-19, predicting critical illness, and assessing prognosis (Li et al., 2020; Zhang et al., 2020). QCT provides detailed anatomical information on pulmonary lesions and extracts comprehensive body composition parameters, including skeletal muscle mass, visceral fat area, and subcutaneous fat thickness. This technology offers novel insights into predicting metabolic status and immune function. Several studies have demonstrated that QCT-derived body composition parameters are closely associated with disease severity and adverse outcomes (Petersen et al., 2020; Hocaoglu et al., 2021; Meyer, Wienke & Surov, 2022; Van Bakel et al., 2023). Meyer, Wienke & Surov (2022) demonstrated that CT-defined low skeletal muscle mass and high visceral adipose tissue significantly impact COVID-19 in-hospital mortality and adverse outcomes (Van Bakel et al., 2023). Additionally, Pranata et al. provided evidence that visceral adipose may influence COVID-19 severity and progression *via* metabolic and inflammatory pathways (Pranata et al., 2021). These findings underscore that sarcopenia and elevated visceral fat are strongly correlated with increased severity and mortality in viral pneumonia patients. Although these studies have preliminarily validated the clinical utility of QCT-derived body composition parameters, the majority of them have focused on single parameters or specific cohorts, lacking integrated analyses of multiple parameters and their interactions. Furthermore, systematic studies exploring the predictive value of QCT-derived body composition for predicting severity and prognosis in viral pneumonia, particularly non-COVID-19 infections, remain scarce. This gap is particularly critical in the post-COVID-19 era, which is characterized by rising trends of co-infections that compound the clinical burden.

Therefore, this study employs a QCT system to assess body composition parameters in viral pneumonia patients, integrating clinical risk factors to predict disease severity and short-term prognoses. The novelty of this study lies in its multi-parameter combined analysis, which elucidates the clinical significance of QCT-derived body composition in viral pneumonia, thereby providing a foundation for precise patient stratification and personalized interventions.

## Materials and Methods

### Study population

This retrospective, single-center, observational study was approved by the Medical Ethics Committee of Fushun Central hospital (zxyyll2023010), and informed consent was waived.

**Figure 1:**
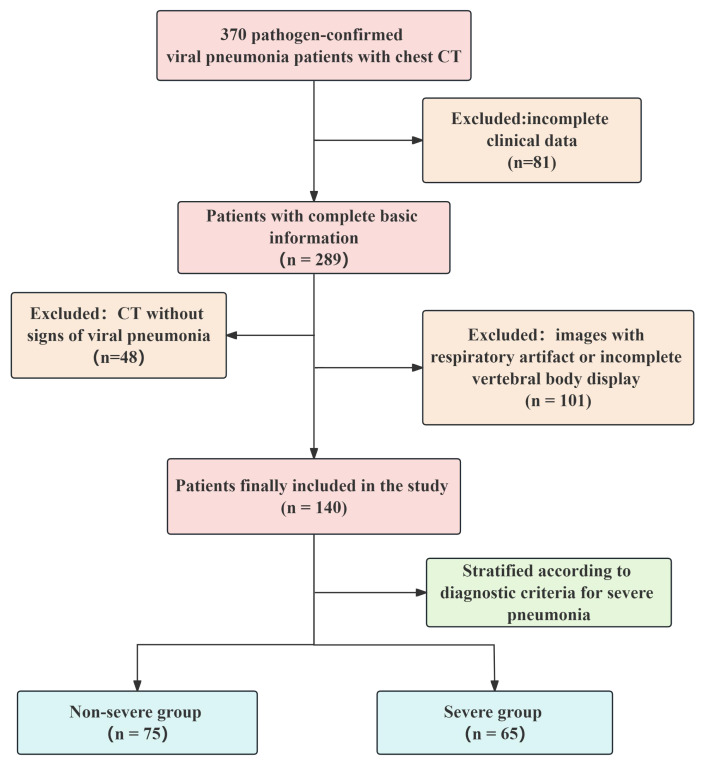
Patient enrollment process.

A total of 140 patients hospitalized for viral pneumonia from December 2022 to January 2024 were included. The inclusion process is shown in Fig. 1. The inclusion criteria were as follows: confirmed viral infection *via* a respiratory viral panel (six-virus panel) that included influenza A, influenza B, respiratory syncytial virus, human rhinovirus, human parainfluenza virus, and adenovirus, a chest CT indicative of viral pneumonia, complete clinical and radiological data, and age ≥ 18 years. The exclusion criteria were as follows: pregnancy and artifacts affecting image quality. Based on the severe pneumonia criteria (Torres et al., 2021), patients were categorized into (1) a control group of non-severe pneumonia patients (*n* = 75) and (2) an experimental group of severe cases (*n* = 65). Adverse outcomes within 30 days of admission were recorded. The severe group was further divided into subgroups with favorable or worsened outcomes, and the parameters were compared. An adverse outcome was defined as the presence of at least one of the following: (1) admission to the ICU, (2) mechanical ventilation, or (3) death.

### Data collection

The clinical data used in this study were extracted from the hospital electronic medical record system, including age, gender, body mass index (BMI), comorbidity, oxygen requirement, length of hospital stay, and laboratory parameters. To ensure the accuracy of the data, all information was independently entered by two physicians and cross-verified.

### CT image acquisition

This study employed either a NeuViz 128-slice CT scanner (Neusoft Medical Systems, Shenyang, China) or a Philips Brilliance 128-slice CT scanner (Philips, Amsterdam, Netherlands) for image acquisition. Chest CT scans were performed using the following parameters: detector configuration, 0.625 mm × 64; tube voltage, 120 kV; automatic tube current modulation; and pitch, 1.0. Patients were placed in the supine position with both upper limbs elevated. The scan range extended from the thoracic inlet to the lower margin of the costophrenic angle to ensure complete coverage of the entire lung parenchyma.

### Quantitative CT analysis of body composition

**Figure 2:**
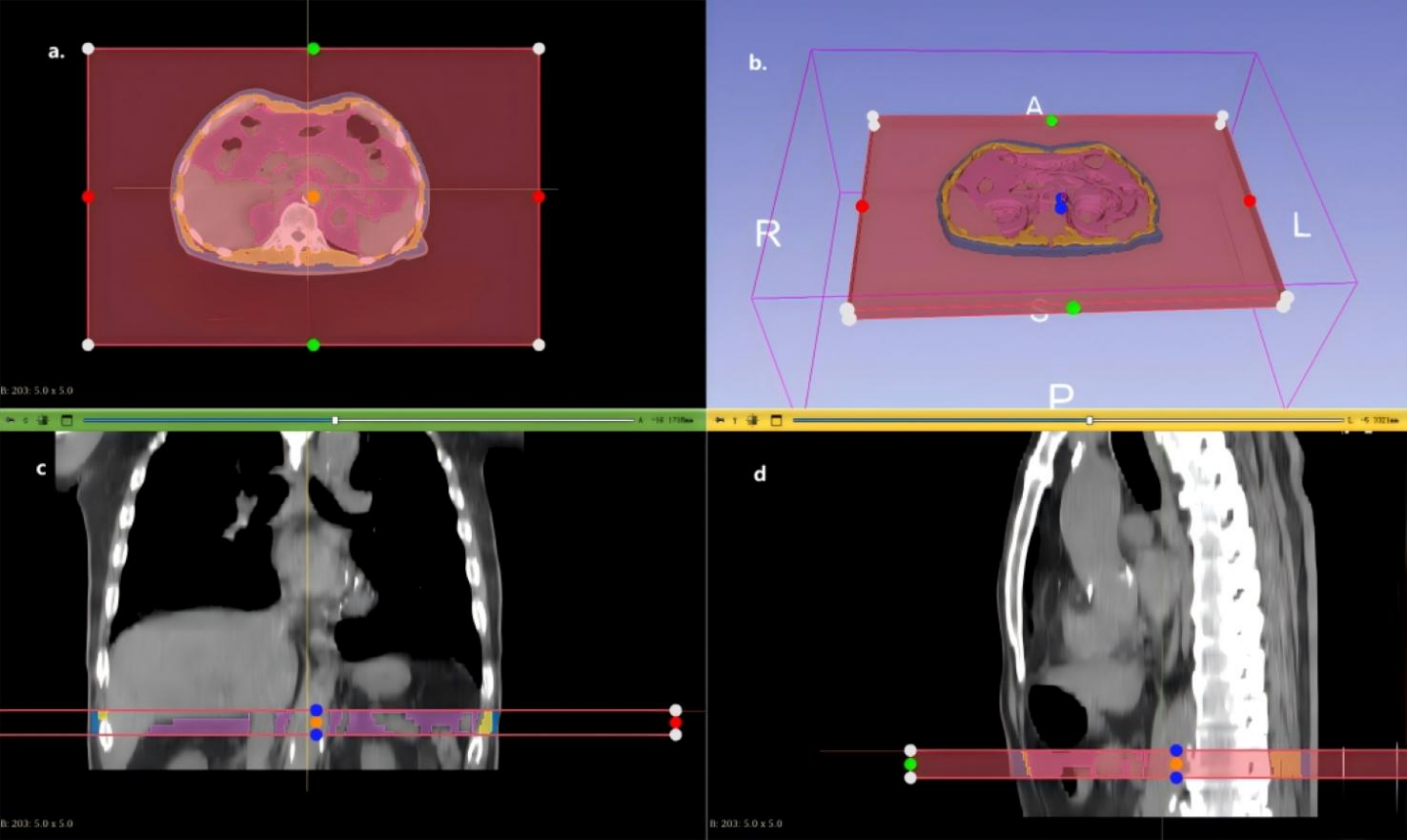
The measurement of body composition on chest CT in a 77-year-old female patient with viral pneumonia. (A) T12 costovertebral joints and (B) all vertebra. In the axial image localisation, the region of interest (ROI) of T12 corresponds to (C) the coronal and (D) sagittal reconstruction images. The blue area represents subcutaneous fat, the purple area represents visceral fat, and the yellow area represents the muscle.

Digital imaging and communications in medicine (DICOM) images with a mediastinal window (slice thickness: five mm; window width, 400 HU; window level: 40 HU) were extracted from the baseline chest CT scans of patients assessed for pneumonia. All imaging data were anonymized and randomly numbered. Two radiologists with over 10 years of experience utilized 3D-Slicer software (*version 5.2.1, https://www.slicer.org/*) to semi-automatically segment muscle and adipose tissues, setting thresholds at −29 to 150 HU for muscle and −150 to −30 HU for fat (Wegener, Fassel & Welger, 1993; Yang et al., 2020). For regions with insufficient separation, manual delineation was performed to ensure precision. Quantitative measurements were taken of the volumes and densities of muscle and adipose tissue at the 4th thoracic (T4), 8th thoracic (T8), and 12th thoracic (T12) costovertebral joints (Fig. 2A) and corresponding whole-vertebral levels (Fig. 2B). T4 measurements included total muscle, peri-thoracic muscle, pectoral muscle, and subcutaneous fat, and T8 and T12 measurements included total muscle, erector spinae muscle, subcutaneous fat, and visceral fat. The boundaries of the T4 and T8 whole vertebrae were defined by costovertebral joints, while T12’s lower boundary was defined at the thoracolumbar junction using facet joints. In axial image localization (Fig. 2A), T12’s region of interest corresponded to the three-dimensional image, as well as the sagittal and coronal reconstruction images (Figs. 2B, 2C and 2D). The number of vertebral image slices was recorded, and the adipose and muscle tissue volumes were divided by the number of vertebral slices for standardization (Lee et al., 2018); that is, the standardized muscle volume (MVs) was calculated as MVs = MVw/(vertebral slice number), where MVw represents the whole-vertebral muscle volume. To offset height variations, the body composition volume measured at the costovertebral joint level was normalized by dividing by the height squared to obtain the body composition index. In other words, the total muscle index (TMI) was calculated as TMI = TMV/ height^2^, where TMV represents the total muscle volume.

### Statistical analysis

Categorical variables were expressed as frequencies and percentages. The Kolmogorov–Smirnov test was used to assess the normality of quantitative data. Data with normal distribution are presented as means ± standard deviations, and non-normally distributed data are reported as median (interquartile range). Between-group comparisons for categorical variables were conducted using the chi-square test. For continuous variables, comparisons were made using the independent-samples *t*-test or the Mann–Whitney U test, depending on their distribution. Spearman’s correlation coefficient (r) was used to evaluate relationships among body composition parameters and between body composition and serological indicators. Univariate and multivariate binary logistic regression analyses identified independent risk factors and were used to develop predictive models: a clinical model, four imaging models (single-slice, whole-vertebral level, standardized whole-layer, and height-normalized body composition models) and a combined model that integrated clinical and imaging parameters. The combined models (1–4) incorporated the clinical model with each of the four imaging models. Model performance was evaluated using receiver operating characteristic (ROC) curves, with the area under the curve (AUC) calculated for each model. Pairwise comparisons of AUCs were conducted using the DeLong test to determine the best predictive model for disease severity. Furthermore, the performance of the optimal model was evaluated using the Hosmer–Lemeshow goodness-of-fit test and a calibration plot to assess calibration. When constructing the calibration plot, the predicted probabilities were grouped into deciles, and the observed event rate in each decile was plotted against the corresponding mean predicted probability. A diagonal line (y = x) was added to the plot to indicate perfect calibration. To evaluate the model’s stability and risk of overfitting, 5-fold cross-validation was performed as internal validation. A *p*-value < 0.05 was considered statistically significant. Statistical analyses were performed using IBM SPSS 26.0 and R (version 4.1).

## Results

### Demographic characteristics

Of the 140 patients, 91 (65%) were male and 65 (46.4%) had severe pneumonia. The median age was 77 years (IQR: 68–85), with a median hospital stay of 10 days. Within 30 days, 26 patients experienced severe adverse outcomes; 24 (92.3%) were from the severe group, and 22 (91.7%) were male. A total of 118 patients (84.3%) had comorbidities, with hypertension (44.3%) being most prevalent, followed by coronary heart disease (35.3%), diabetes (25.2%), and cerebrovascular diseases (23.6%) (Table 1). The severe group was significantly older (*p* = 0.023), had higher oxygen requirements (*p* < 0.001), and had a greater incidence of 30-day adverse outcomes (*p* < 0.001) than the non-severe group. However, there were no significant inter-group differences in gender, BMI, or hospital stay duration (*p* > 0.05). Inflammatory markers (Neu%, CRP, and procalcitonin) were significantly elevated in the severe group (*p* < 0.001), while the lymphocyte (Lym%) and eosinophil (Eos%) percentages were lower (*p* < 0.01).

**Table 1:** Demographic characteristics.

Indicator	All (*n*= 140)	Non-severe (*n*= 75)	Severe (*n*= 65)	*p*-value
Age (years)*(mean*±*SD or median (IQR))*	77 (68–85)	76 (67–83)	82 (70–87)	0.023
Sex, n	91 male 49 women	45 male 30 women	46 male 19 women	0.184
Height (m) *(mean*±*SD or median (IQR))*	1.70 (1.60–1.73)	1.68 (1.60–1.73)	1.70 (1.60–1.75)	0.453
Weight (kg) *(mean*±*SD or median (IQR))*	65.0 (60.0–70.0)	65.0 (56.3–70.0)	65.0 (60.0–75.0)	0.168
BMI (kg/m^2^) *(mean*±*SD or median (IQR))*	23.16 (20.77–25.11)	22.86 (20.02–24.74)	23.43 (21.35–25.71)	0.200
Oxygen supply, yes n (%)	101 (72.1)	40 (53.3)	61 (93.8)	<0.001
Length of hospital stay (days) (*median (IQR))*	10 (8–14)	11 (8–14)	10 (6.5–14)	0.352
Overall comorbidity n(%)	118 (84.3)	63 (84.0)	55 (84.6)	0.921
Adverse outcome, yes n(%)	26 (18.6)	2 (2.7)	24 (36.9)	<0.001
Lym% *(median (IQR))*	11.05 (6.83–18.48)	15.10 (8.75–21.45)	9.20 (5.23–13.80)	<0.001
Neu% *(mean*±*SD or median (IQR))*	81.70 (71.93–87.80)	75.94 (68.23–84.48)	85.30 (77.78–90.30)	<0.001
Eos% (*median (IQR))*	0.15 (0.00–0.60)	0.20 (0.03–0.70)	0.10 (0.00–0.48)	0.002
WBC (10^9^/L)(*median (IQR))*	7.17 (5.20–9.94)	6.10 (4.90–8.98)	8.32 (6.02–11.15)	0.003
CRP (mg/L) (*median (IQR))*	72.49 (27.01–153.17)	39.25 (16.77–97.12)	110.31 (57.82–197.02)	<0.001
PCT (ng/ml) (*median (IQR))*	0.204 (0.17–0.54)	0.17 (0.11–0.22)	0.25 (0.20–2.17)	<0.001

**Notes.**

Quantitative variables are presented as the mean ± standard deviation for normally distributed datasets and as the median (interquartile range) for non-normally distributed datasets. Qualitative variables are denoted by patient counts, with percentages shown in parentheses.

Abbreviations BMI body mass index Lym% percentage of lymphocytes Neu% percentage of neutrophils Eos% percentage of eosinophils CRP C-reactive protein PCT procalcitonin

### Comparison of body composition between the severe and non-severe groups

#### Relationship between single-slice body composition at costovertebral joint level and disease severity

At T4 and T8, neither adipose nor muscle tissues were associated with disease severity (all *p* > 0.05) (Table S1). In contrast, the severe group had a lower T12-ESV (*p* = 0.045), T12-ESD (*p* = 0.046), and higher T12-VAV (*p* = 0.006) than the non-severe group; however, no significant difference was found in T12-SAV (*p* = 0.895) (Table 2).

**Table 2:** Comparison of body composition between severe and non-severe patients (mean ± SD or median (IQR)).

	All patients (*n*= 140)	Non-Severe (*n*= 75)	Severe (*n*= 65)	*p*-value
a. Body composition at the costovertebral joints level (Single slice measurement)				
T12-TMV	21.66 (17.33–28.62)	21.30 (17.10–30.95)	21.67 (17.74–26.38)	0.424
T12-TMD	27.08 ± 9.61	27.46 ± 10.26	26.65 ± 8.88	0.528
T12-ESV	9.57 (7.54–11.73)	9.98 (7.95–12.45)	9.04 (6.68–11.35)	0.045
T12-ESD	30.12 (21.61–37.77)	32.65 (23.20–39.64)	29.26 (21.30–34.67)	0.046
T12-SAV	26.75 (17.80–43.32)	26.99 (16.59–38.79)	26.75 (18.30–45.52)	0.859
T12-SAD	−86.39 (−93.75 to −77.24)	−86.84 (−93.69 to −77.26)	−85.84 (−95.56 to −76.60)	0.928
T12-VAV	40.77 (23.59–63.00)	33.03 (19.46–54.64)	45.88 (30.78–71.01)	0.006
T12-VAD	−82.74 (−88.38 to −76.59)	−81.67 (−86.86 to −76.14)	−84.58 (−89.52 to −76.86)	0.134
b. Body composition at the whole vertebral levels				
T8-VAVw	46.45 (30.87–65.23)	43.12 (24.94–87.81)	51.10 (35.65–66.53)	0.015
T8-VADw	−76.21 (−80.12 to −69.52)	−76.14 (−80.26 to −68.86)	−76.27 (−79.76 to −70.19)	0.872
T12-TMVw	141.40 (102.24–200.59)	143.30 (104.96–200.59)	138.23 (97.37–201.68)	0.641
T12-TMDw	26.97 ± 9.50	27.40 ± 9.96	26.49 ± 9.01	0.482
T12-ESVw	58.85 (45.91–76.43)	59.64 (45.91–85.13)	58.14 (44.87–73.19)	0.338
T12-ESDw	28.88 (21.32–37.18)	33.25 (22.15–38.15)	27.20 (20.80–34.21)	0.030
T12-SAVw	166.40 (107.39–266.59)	154.30 (102.88–233.90)	173.70 (108.61–276.57)	0.395
T12-SADw	−86.62 (−93.68 to −78.80)	−87.20 (−93.68 to −79.49)	−86.19 (−94.77 to −76.80)	0.975
T12-VAVw	257.90 (152.74–401.79)	225.60 (121.62–319.76)	300.30 (222.40–452.15)	0.005
T12-VADw	−83.72 (−88.33 to −77.54)	−82.80 (−87.24 to −77.03)	−85.27 (−90.04 to −77.62)	0.255
c. Standardised body composition (measurements at the whole vertebral level/vertebral slice number)				
T12-TMVs	30.37 (24.48–37.32)	31.33 (25.52–37.61)	29.05 (23.82–37.26)	0.186
T12-ESVs	12.46 (10.33–14.89)	12.98 (10.90–15.74)	11.90 (9.90–14.35)	0.023
T12-SAVs	35.51 (23.16–53.10)	35.80 (22.72–52.20)	35.46 (23.26–55.84)	0.626
T12-VAVs	58.58 ± 29.78	51.62 ± 27.75	66.42 ± 30.25	0.006
d. Body composition index(measurements at the costovertebral joints level/height^2^)				
T12-TMI	7.63 (6.35–10.22)	7.57 (6.24–14.16)	7.82 (6.46–9.36)	0.393
T12-ESI	3.39 (2.66–4.25)	3.49 (2.92–4.39)	3.18 (2.47–4.13)	0.027
T12-SAI	9.52 (6.61–15.37)	9.57 (5.86–14.69)	9.02 (6.73–15.79)	0.992
T12-VAI	14.71 (8.24–23.55)	11.79 (6.96–20.18)	16.09 (10.77–24.92)	0.006

**Notes.**

Quantitative variables are presented as the mean ± standard deviation for normally distributed datasets and as the median (interquartile range) for non-normally distributed datasets.

Abbreviations TMV total muscle volume TMD total muscle density ESV erector spinae volume ESD erector spinae density SAV subcutaneous adipose volume SAD subcutaneous adipose density VAV visceral adipose volume VAD visceral adipose density -w body composition measured at the whole vertebral level -s standardized body composition (measurements at the whole vertebral level/vertebral slice number) -I body composition index (single layer of body composition volume/height^2^)

#### Relationship between whole-vertebral level body composition and disease severity

Unlike single-slice QCT, T8-VAVw showed a statistically significant difference between the two groups (*p* = 0.015) (Table 2), whereas no significant differences were observed in the body composition at the T4 whole-vertebral level and in the remaining parameters at the T8 whole-vertebral level (all *p* > 0.05) (Table S1). However, patients in the severe group had significantly lower T12-ESDw (*p* = 0.030) and significantly higher T12-VAVw (*p* = 0.005) than those in the non-severe group, while T12-ESVw and T12-SAVw showed no significant differences (*p* > 0.3) (Table 2).

#### Relationship between standardized body composition volumes and disease severity

No significant differences were found in the standardized body composition at T4 and T8 between the two groups (all *p* > 0.05) (Table S1). The severe group had lower T12-ESVs than those of the non-severe group (*p* = 0.023), showing a more significant difference than the comparison for T12-ESV (*p* = 0.045). T12-VAVs was significantly higher in the severe group than in the non-severe group (*p* = 0.006). However, no significant difference was found in T12-SAVs between the two groups (*p* = 0.626) (Table 2).

#### Relationship between body composition index and disease severity

The body composition index (BCI) was calculated by normalizing the single-layer body composition volume by height. At T4 and T8, BCI showed no significant differences between the two groups (all *p* > 0.05) (Table S1). In contrast, T12-ESI and T12-VAI demonstrated significant differences (*p* = 0.027 and *p* = 0.006, respectively) (Table 2).

#### Body composition comparison between patients with and without 30-day adverse outcomes in the severe group

**Figure 3:**
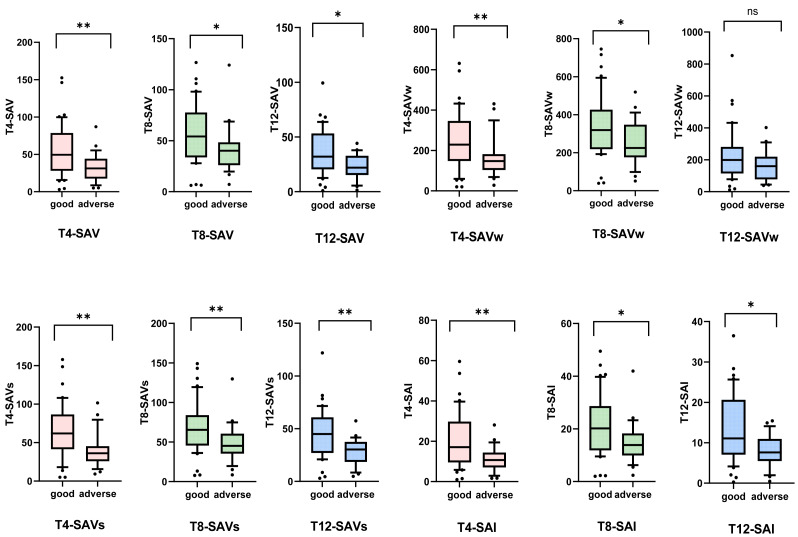
Differences in subcutaneous fat and its parameters among viral pneumonia patients with different outcomes. Box plots represent the distribution of subcutaneous fat and its related parameters among viral pneumonia patients with different clinical outcomes. The box spans the interquartile range (IQR, Q1–Q3), with the horizontal line indicating the median. Whiskers represent the 10th and 90th percentiles, and dots outside the whiskers denote outliers. * represents *P* < 0.05; ** represents *P* < 0.01; ns: not significant Abbreviations: SAV, subcutaneous adipose volume; -w, body composition measured at the whole vertebral level; -s, standardized body composition (measurements at the whole vertebral level/vertebral slice number); -I, body composition index (single layer of body composition volume/height^2^).

**Figure 4:**
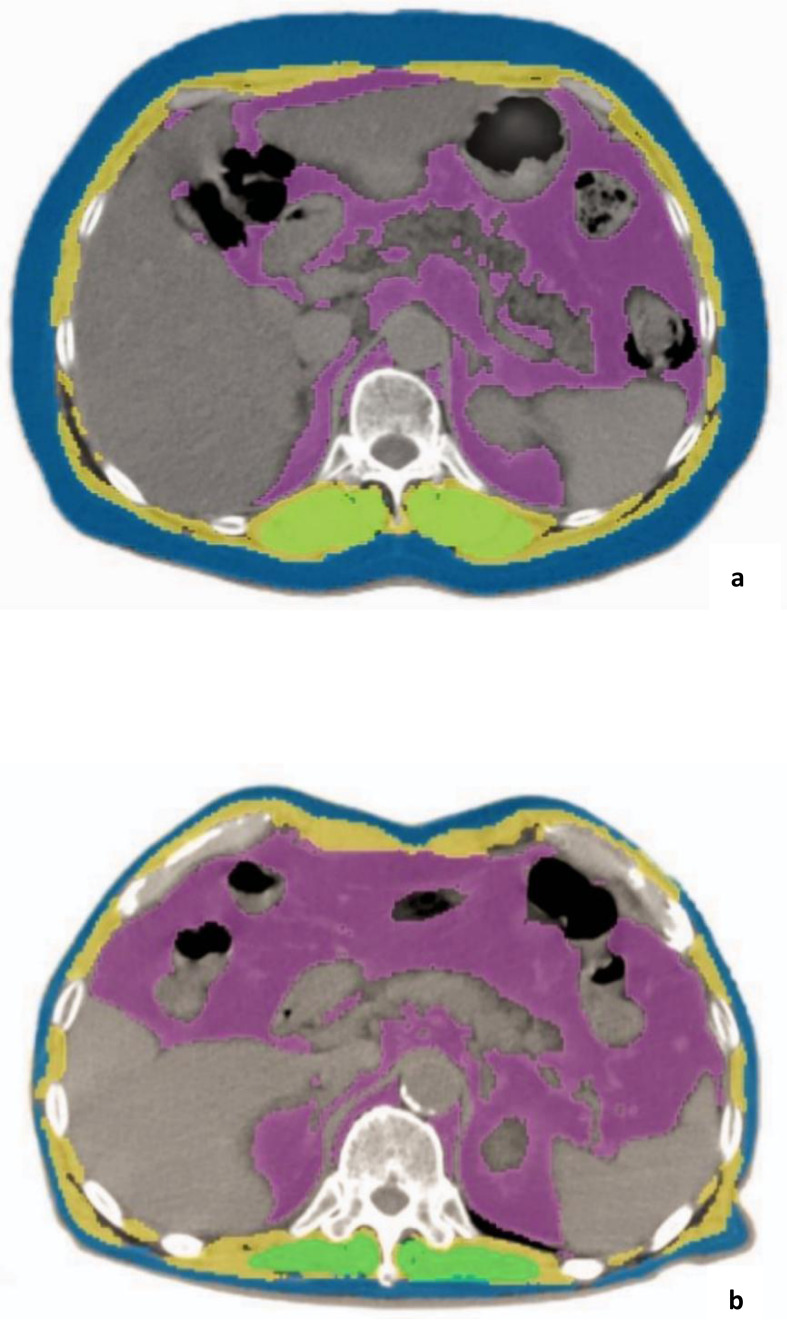
Representative cases showing the comparison of adipose tissue and skeletal muscle distribution at T12 between the severe and non-severe groups. (A) Female, 69 years old, BMI 24.6 kg/m^2^, non-severe case with favourable outcome. Axial image at the T12 costovertebral joint level with quantified SAV (blue) = 65.6 cm^3^, VAV (purple) = 49.6 cm^3^, TMV (yellow) = 33.2 cm^3^, and ESV (green) = 10.1 cm^3^. (B) Male, 71 years old, BMI 24.2 kg/m^2^, severe case with adverse outcome. Axial image at T12 costovertebral joint level with quantified SAV (blue) = 16.9 cm^3^, VAV (purple) = 93.4 cm^3^, TMV (yellow) = 30.5 cm^3^, and ESV (orange) = 7.6 cm^3^. Abbreviations: SAV, subcutaneous adipose volume; VAV, visceral adipose volume; TMV, total muscle volume; ESV, erector spinae volume.

A total of 24 patients (36.9%) in the severe group experienced adverse outcomes within 30 days. Adverse outcomes were predominantly noted in elders (*p* = 0.004) and males (*p* = 0.005), who exhibited notably less subcutaneous fat (*p*_T4-SAV_ = 0.004, *p*_T4-SAVw_ = 0.008, *p*_T4-SAVs_ = 0.002; *p*_T8-SAV_ = 0.021, *p*_T8-SAVw_ = 0.026, *p*_T8-SAVs_ = 0.004; *p*_T12-SAV_ = 0.020, *p*_T12-SAVs_ = 0.002) (Table 3). However, neither visceral adipose volume nor muscle volume was associated with clinical outcomes (all *p* > 0.05) (Table S2). Figure 3 shows the differences in the subcutaneous fat parameters at different thoracic vertebral levels among viral pneumonia patients with diverse clinical outcomes. Figure 4 compares the adipose and muscle tissue distribution at T12 in patients with varying severities and clinical outcomes.

**Table 3:** Comparison of body composition in 65 severe patients with adverse outcomes or not (mean ± SD or median (IQR)).

	Severe (*n*= 65)	Good (*n*= 41)	Adverse (*n*= 24)	*p*-value
a. Body composition at the costovertebral joints level (Single slice measurement)				
T4-SAV	42.73 (24.04–59.46)	49.37 (27.81–78.98)	31.32 (17.62–44.25)	0.004
T4-SAD	−85.89 (−95.99 to −78.52)	−91.00 (−98.64 to −81.61)	−79.96 (−86.75 to −74.21)	0.001
T8-SAV	43.20 (30.92–67.52)	54.10 (33.63–77.61)	40.12 (26.04–48.45)	0.021
T8-SAD	−89.18 (−95.91 to −80.38)	−89.50 (−97.52 to −82.95)	−85.20 (−92.90 to −67.19)	0.055
T12-SAV	26.75 (18.30–45.52)	32.04 (20.43–53.35)	21.98 (15.21–32.85)	0.020
T12-SAD	−85.84 (−95.56 to −76.60)	−88.93 (−99.45 to −81.20)	−82.73 (−87.45 to −65.70)	0.007
b. Body composition at the whole vertebral levels				
T4-SAVw	181.10 (122.25–299.34)	228.92 (147.57–346.13)	147.86 (103.06–182.19)	0.008
T4-SADw	−85.94 (−96.85 to −79.75)	−92.30 (−98.83 to −81.00)	−81.67 (−85.95 to −72.88)	0.001
T8-SAVw	275.10 (201.59–386.25)	319.21 (218.94–426.68)	224.75 (175.90–347.45)	0.026
T8-SADw	−87.60 (−95.95 to −80.05)	−90.45 (−96.77 to −81.79)	−82.80 (−92.56 to −67.00)	0.017
T12-SAVw	173.70 (108.61–276.57)	200.31 (113.65–282.03)	159.95 (77.45–220.10)	0.118
T12-SADw	−86.19 (−94.77 to −76.80)	−89.15 (−99.77 to −81.78)	−83.66 (−87.96 to −66.06)	0.006
c. Standardised body composition (measurements at the whole vertebral level/vertebral slice number)				
T4-SAVs	46.04 (30.56–76.09)	61.93 (41.42–86.53)	36.15 (25.77–45.39)	0.002
T8-SAVs	54.28 (41.08–77.25)	65.44 (45.48–84.05)	45.23 (35.42–60.65)	0.004
T12-SAVs	35.46 (23.26–55.84)	44.90 (26.93–60.76)	30.28 (18.46–37.58)	0.002
d. Body composition index(measurements at the costovertebral joints level/height^2^)				
T4-SAI	14.39 (9.07–21.66)	16.91 (9.43–29.82)	10.71 (7.04–14.42)	0.004
T8-SAI	15.39 (10.84–24.88)	20.18 (11.82–28.64)	13.89 (9.87–18.27)	0.026
T12-SAI	9.02 (6.73–15.79)	11.30 (6.90–20.82)	7.60 (5.45–10.98)	0.018

**Notes.**

Short-term adverse outcomes included admission to the intensive care unit, mechanical ventilation, or death within 30 days of disease onset. Quantitative variables are presented as the mean ± standard deviation for normally distributed datasets and as the median (interquartile range) for non-normally distributed datasets. Qualitative variables are denoted by patient counts, with percentages indicated in parentheses.

Abbreviations SAV subcutaneous adipose volume SAD subcutaneous adipose density -w body composition measured at the whole vertebral level -s standardized body composition (measurements at the whole vertebral level/vertebral slice number) -I body composition index (single layer of body composition volume/height^2^)

### Correlation analysis between predictors

**Figure 5:**
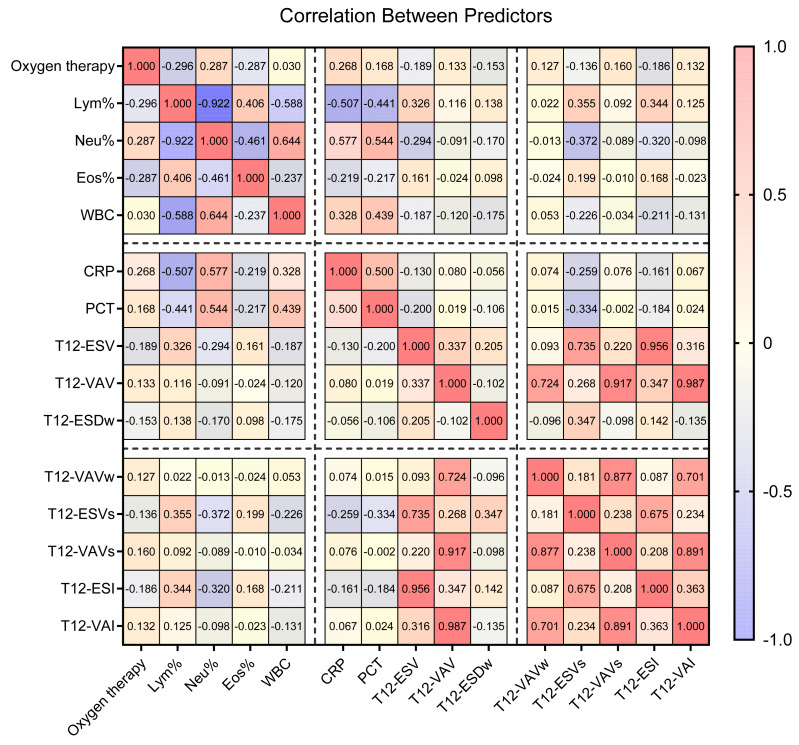
Correlation analysis between predictors. Spearman’s correlation coefficient (r) is indicated in the boxes. Abbreviations: Lym%, percentage of lymphocytes; Neo%, percentage of neutrophils; Eos%, percentage of eosinophils; CRP, C-reactive protein; PCT, procalcitonin. ESV, erector spinae volume; ESD, erector spinae density; VAV, visceral adipose volume; -w, body composition measured at the whole vertebral level; -s, standardized body composition (measurements at the whole vertebral level/vertebral slice number); -I, body composition index (single layer of body composition volume/height^2^).

Spearman’s correlation analysis was conducted to further explore the relationships among predictive factors, and this indicated positive correlations of T12-ESV with T12-ESVs and T12-ESI (*r* = 0.735, *r* = 0.956, respectively; *p* < 0.001) (Fig. 5). Similarly, T12-VAV was positively correlated with T12-VAVw, T12-VAVs, and T12-VAI (*r* = 0.724, *r* = 0.917, *r* = 0.987, *p* < 0.001). Furthermore, Lym% was correlated with T12-ESV, T12-ESVs, and T12-ESI (*r* = 0.326, *r* = 0.355, *r* = 0.344, *p* < 0.001). However, T12-VAV, T12-VAVs, T12-VAVw, and T12-VAI showed no correlations with laboratory markers (all *p* > 0.05). Notably, Lym% showed moderate and very strong negative correlations with the CRP count and Neu% (*r* = −0.507, *r* = −0.922, *p* < 0.001).

### Univariate and multivariate logistic regression analyses of risk factors for disease severity

The univariate analysis included age, oxygen therapy, laboratory indices, and body composition variables that were significant in between-group com*p* arisons (*p* < 0.05) (Table 4). Variables that were significant in the univariate logistic regression (*p* < 0.05) were then included in the multivariate analysis. The results indicated that CRP (OR = 1.006, 95% CI [1.000–1.012]), oxygen demand (OR = 10.934, 95% CI [3.517–33.987]), T12-VAV (OR = 1.030, 95% CI [1.014–1.047]), T12-VAVw (OR = 1.003, 95% CI [1.000–1.005]), T12-VAVs (OR = 1.021, 95% CI [1.009–1.034]), and T12-VAI (OR = 1.088, 95% CI [1.040–1.139]) were independent risk factors for viral pneumonia severity. Conversely, T12-ESV (OR = 0.823, 95% CI [0.733–0.924]), T12-ESDw (OR = 0.971, 95% CI [0.944–0.999]), T12-ESVs (OR = 0.860, 95% CI [0.779–0.950]), and T12-ESI (OR = 0.569, 95% CI [0.415–0.781]) were found to be independent protective factors. All of these variables were significant predictors of disease severity (all *p* < 0.05).

**Table 4:** Univariate and multivariate analysis of risk factors for the severity of viral pneumonia.

Indicator	Univariate			Multivariate			
	*OR*	95% *CI*	*p*-value	*OR*	95% *CI*	*p*-value
**Model 1**						
Age	1.032	1.000–1.064	0.046	1.015	0.980–1.051	0.407
Lym%	0.946	0.907–0.986	0.009	1.007	0.964–1.051	0.770
Eos%	0.379	0.188–0.761	0.006	0.523	0.253–1.079	0.079
WBC	1.122	1.030–1.222	0.008	1.050	0.958–1.152	0.298
CRP	1.009	1.005–1.014	<0.001	1.006	1.001–1.012	0.030
PCT	1.177	1.029–1.347	0.018	1.064	0.927–1.222	0.378
**Model 2**						
CRP	1.009	1.005–1.014	<0.001	1.008	1.003–1.013	0.003
Oxygen therapy	13.344	4.403–40.436	<0.001	10.934	3.517–33.987	<0.001
**Model 3**						
T12-ESV	0.895	0.816–0.980	0.017	0.823	0.733–0.924	0.001
T12-ESD	0.977	0.951–1.003	0.076	0.991	0.963–1.019	0.521
T12-VAV	1.018	1.005–1.031	0.008	1.030	1.014–1.047	<0.001
**Model 4**						
T8-VAVw	1.010	0.998–1.022	0.101	1.000	0.987–1.014	0.968
T12-ESDw	0.971	0.945–0.999	0.039	0.971	0.944–0.999	0.041
T12-VAVw	1.003	1.001–1.005	0.006	1.003	1.000–1.005	0.024
**Model 5**						
T12-ESVs	0.904	0.828–0.987	0.024	0.860	0.779–0.950	0.003
T12-VAVs	1.015	1.004–1.027	0.008	1.021	1.009–1.034	0.001
**Model 6**						
T12-ESI	0.736	0.569–0.951	0.019	0.569	0.415–0.781	<0.001
T12-VAI	1.049	1.012–1.088	0.009	1.088	1.040–1.139	<0.001

**Notes.**

After excluding confounding factors such as collinearity, the statistically significant variables between groups were incorporated into univariate logistic regression analysis, followed by multivariate analysis using various combination models. A bilateral *P* < 0.05 was considered statistically significant.

Abbreviations Lym% percentage of lymphocytes Eos% percentage of eosinophils CRP C-reactive protein PCT procalcitonin ESV erector spinae volume ESD erector spinae density VAV visceral adipose volume VAD visceral adipose density -w body composition measured at whole vertebral level -s standardized body composition (measurements at the whole vertebral level/vertebral slice number) -I body composition index (single layer of body composition volume/height^2^)

### Models for predicting the severity of viral pneumonia

**Figure 6:**
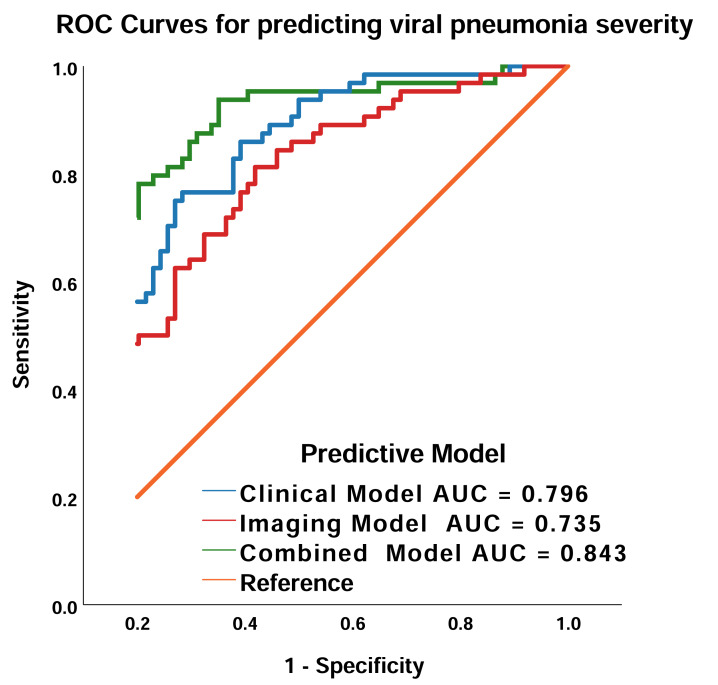
Receiver operating characteristic (ROC) curve analysis for prediction of severe viral pneumonia. Clinical model (oxygen therapy and CRP; AUC, 0.796; 95% CI [0.723–0.870]), optimal imaging model (T12-ESV and T12-VAV; AUC, 0.735, 95% CI [0.651–0.815]), and a combined model consisting of the above two models (oxygen therapy, CRP, T12-ESV, and T12-VAV; AUC, 0.843, 95% CI [0.778–0.909]). The combined model had the highest predictive value.

The clinical model that incorporated oxygen therapy and CRP exhibited an AUC of 0.796 (95% CI [0.723–0.870]). Although the differences in AUC between the four imaging models were not statistically significant, the imaging model 1 (T12-ESV and T12-VAV) achieved the highest AUC (0.735, 95% CI [0.651–0.815]) and was identified as the optimal imaging model. The combined model integrating the clinical model (oxygen therapy, CRP) and the imaging model 1 (T12-ESV, T12-VAV) demonstrated good discriminative ability, with an AUC of 0.843 (95% CI [0.778–0.909]). This model significantly outperformed both the imaging model 1 and the clinical model (*p* < 0.001) (Table S3 and Fig. 6). The Hosmer–Lemeshow test yielded a chi-square value of 8.71 (degrees of freedom = 8, *p* = 0.367), indicating a good model fit. The calibration plot showed acceptable agreement between the predicted and observed probabilities across most risk quantile intervals, although a slight deviation was observed in the intermediate-risk range (Fig. S1). The mean AUC obtained from 5-fold cross-validation was 0.859 (SD = 0.032), suggesting a low risk of overfitting (Fig. S2).

## Discussion

This study investigated the value of chest CT-derived body composition parameters and clinical risk factors in predicting viral pneumonia severity. Patients were categorized into severe and non-severe groups, and a subgroup analysis of 30-day adverse outcomes was performed in the severe group. Although both the clinical and imaging models demonstrated good predictive performance, the combined model showed improved accuracy, as evidenced by an increase in the AUC from 0.796 and 0.735 to 0.843. This finding highlighted that imaging-derived body composition can be effectively combined with clinical factors to identify severe cases.

Different imaging modalities for assessing body composition vary in their characteristics. While magnetic resonance imaging involves no radiation and can accurately quantify muscle and fat, its high cost and lengthy scan time ensure that it is seldom used in the routine management of acute pneumonia. Ultrasonography is portable and non-irradiating, making it suitable for the bedside assessment of diaphragmatic function; however it is inadequate for comprehensive and reproducible whole-body quantitative body composition analysis. In contrast, CT serves as a standard modality for evaluating the severity and extent of pneumonia, possessing broad clinical applications. Nevertheless, the value of utilizing these readily acquired chest CT images for in-depth body composition analysis to predict the severity and prognosis of viral pneumonia has not been fully explored, with a particular lack of studies that systematically compare different thoracic vertebral levels. Therefore, this study systematically analyzed body composition parameters at the T4, T8, and T12 levels from baseline chest CT scans, including single-layer, whole-vertebral, standardized, and height-normalized metrics (Van Ee et al., 2024), to evaluate disease severity and short-term prognosis. Its key innovation lies in integrating visceral adipose and skeletal muscle mass data obtained from different layers of routine CT scans to establish a more comprehensive predictive model for viral pneumonia severity and prognosis compared to single-parameter assessment. The results indicated that elderly patients with higher T12-VAV, lower T12-ESV, and lower T12-ESD were at greater risk of developing severe conditions. Furthermore, lower SAV was associated with poorer outcomes, while elderly males faced higher 30-day adverse event risks. To control for potential confounding influences of height, body type, age, nutritional status, and physical activity, this study employed standardized and height-normalized body composition parameters.

The combined model (incorporating oxygen therapy, CRP, T12-ESV, T12-VAV) achieved the highest AUC for severity prediction. The calibration curve exhibited a slight underestimation of risk in the low-to-intermediate predicted probability range and a slight overestimation in the high predicted probability range, which may be attributable to the limited sample size or the presence of unmeasured confounders. Nevertheless, the Hosmer–Lemeshow test was not statistically significant, indicating that the overall calibration was acceptable. External validation in a larger future cohort is warranted to confirm these findings. Imaging models based on different quantitative methods showed no significant difference in predicting viral pneumonia. Therefore, when stratifying patients, simple and accessible single-layer body composition quantification methods should be prioritized. The simplicity and speed of the method can promote its efficient integration into the clinical workflow and allow for rapid assessment. In this study, we utilized the open-source software 3D-Slicer to quantify adipose and muscular parameters from baseline chest CT scans, a routine clinical procedure for assessing pneumonia severity that requires no additional radiation exposure. This approach provided valuable insights into understanding disease progression and facilitated the timely identification of severe cases and adverse outcomes.

This study confirms the independent association of T12-ESV (OR = 0.823) and T12-VAV (OR = 1.030) with pneumonia severity through several interconnected biological pathways. Skeletal muscle serves not only as a respiratory engine but also as a key metabolic and immune-modulatory organ. Reduced muscle mass may lead to: (1) diminished respiratory muscle strength and endurance, impairing ventilation and cough efficacy, thereby increasing the risk of atelectasis and secondary infection; (2) compromised immunity due to protein-energy malnutrition and the decreased synthesis of immunomodulatory myokines (Severinsen & Pedersen, 2020; Rajamanickam et al., 2022); and (3) depleted metabolic reserves, reducing the capacity to cope with infection-induced hypermetabolic stress. Conversely, an abnormal fat distribution-particularly visceral adipose accumulation—exerts pro-inflammatory and metabolically dysregulated effects. As an active endocrine organ, visceral adipose tissue expansion is associated with: (1) systemic chronic low-grade inflammation, where continuously released pro-inflammatory cytokines may synergize with acute infection-triggered cytokine storms, to aggravate tissue injury (Wulandari, Hartono & Wibawa, 2023); (2) insulin resistance and metabolic dysfunction, altering energy homeostasis and immune cell activity; and (3) the mechanical restriction of diaphragmatic movement and lung expansion. Thus, the “low muscle–high visceral fat” phenotype likely defines a vulnerable state comprising high inflammatory load and a low physiological reserve, leaving individuals with limited compensatory capacity during acute insults such as pneumonia and increasing their risk of severe disease. The OR values further quantify these relationships, indicating that greater muscle volume is correlated with lower risk, whereas greater visceral fat volume is correlated with higher risk. These mechanisms provide a solid pathophysiological basis for our imaging findings and highlight the importance of incorporating nutritional and metabolic assessment in clinical evaluation.

The findings of this study are consistent with previous literature while also providing a novel perspective. Previous studies have shown that CT-quantified visceral fat accumulation is an important risk factor for severe COVID-19, offering greater predictive value than subcutaneous fat or BMI (Pranata et al., 2021; Favre et al., 2021; Watanabe et al., 2020). However, its applicability for predicting the severity of other viral pneumonias in the post-pandemic era remains unclear. Moreover, most studies have focused on cross-sectional body composition at the L3 level, which is not applicable to viral pneumonia patients who typically undergo only chest CT scans. Given that body composition at the T12 level is highly correlated with that at L3, we evaluated its reliability as making it a viable alternative indicator (Molwitz et al., 2022), and found that VAV outperformed SAV in predicting disease severity. Severe patients exhibited higher VAV and lower SAV, consistent with prior studies (Petersen et al., 2020; Pranata et al., 2021). The distribution and quantity of adipose tissue significantly affect immunomodulation. Respiratory viruses inhibit lipolysis in subcutaneous fat while upregulating pro-inflammatory factors in visceral fat, linking visceral obesity to severe influenza-related respiratory complications (Saccon et al., 2022). Viral receptor concentrations are higher in visceral fat than in subcutaneous fat, which increases the risk of infection (Gnanenthiran et al., 2022). We also found that patients with lower subcutaneous fat were more prone to adverse outcomes. Moderate subcutaneous fat may confer protection against disease progression. Aging exacerbates ectopic fat accumulation, potentially impairing the immune-regulatory function of visceral fat.

In our study, severe patients had significantly lower ESV and ESD at T12, confirming that their decline is a risk factor for severe viral pneumonia. This result is consistent with prior studies (Hocaoglu et al., 2021; Hosch et al., 2022). Skeletal muscles secrete myokines that support immune cells by supplying amino acids *via* protein metabolism (Wang, Li & Wang, 2021). Patients with sarcopenia are more susceptible to respiratory viral infections due to weakened immunity. Furthermore, sarcopenia reduces respiratory muscle strength and lung capacity, thereby impairing respiratory function and increasing vulnerability to viral pneumonia (Ohara et al., 2018). However, in this study, we found no association between pectoral muscle volume or density at T4 and viral pneumonia severity. This finding contrasts with some prior research (Koehler et al., 2022; Montes-Ibarra et al., 2023). This discrepancy could be attributed to distinct patient characteristics in our retrospective cohort, which included elderly individuals with multiple comorbidities and complex clinical profiles. Additionally, technical factors such as the inability of some participants to fully elevate their arms or maintain the prescribed position during CT acquisition might have led to inadequate contraction or relaxation of the intercostal muscles, potentially affecting T4 measurements. At the T8 level, the predominance of lung tissue may obscure muscle and fat, limiting the predictive value of body composition for disease severity. In contrast, T12 is located at the thoracic-abdominal junction and contains more fat and muscle than T4 or T8, including a higher proportion of visceral (particularly abdominal) fat. Consequently, T12 may be the optimal level for assessing body composition on chest CT. Our results confirmed a significant association between T12-ESV, T12-VAV and their parameters and viral pneumonia severity. These findings offer guidance for selecting appropriate measurement sites and methods on a chest CT to predict the progression and prognosis of other acute pulmonary infections.

In addition to exploring imaging markers, this study also examined the role of conventional inflammatory indicators. The results demonstrated that Lym%, Neu%, CRP, and procalcitonin are significantly associated with the severity of viral pneumonia . T12-ESV and Lym% were moderately correlated, with lower levels of both indicating more severe conditions. Lymphocytes play a key role in immune defense by providing immune protection (Kwiecień et al., 2020). The findings suggested that lymphocyte reduction and sarcopenia may exacerbate disease by mediating immune suppression. However, T12-VAV exhibited weaker correlations with laboratory markers. Furthermore, the strong correlation of T12-ESV and T12-VAV with related parameters further supported the interchangeability of different quantitative methods.

This study has several limitations. First, the sample size was relatively small, and the lack of both an external validation cohort and a control group including patients with other types of pneumonia limits the generalizability of our conclusions. Therefore, further prospective multi-center studies are required to validate these findings and extend their applicability to other acute pulmonary infections. Additionally, the diagnosis of viral pneumonia relied on a six-virus panel that did not cover all potential viral pathogens and could not assess the detection of mixed or rare viral infections, constituting another methodological constraint. Second, as this was a single-center study involving a predominantly elderly population with a high prevalence of comorbidities and few young participants, the results may not be generalizable to younger populations or other clinical settings. Future prospective studies encompassing a broader age range of patients and multiple centers are warranted to validate and extend our findings. Third, in the present study, rigorous paired agreement assessments and comparisons among the different methods for quantifying body composition parameters were not performed; conducting these analyses remains an important direction for future research. In addition, this study carries potential overfitting risks and lacks robustness testing for missing inputs. Finally, there may be a bidirectional effect between sarcopenia and the severity of acute viral pneumonia (Rossi et al., 2022). Nevertheless, by analyzing baseline CT scans obtained early in the infection course, we sought to minimize the potential confounding influence of disease progression on body composition measurements.

## Conclusion

In conclusion, T12-ESV, T12-VAV, and related parameters were found to be significantly correlated with the severity of viral pneumonia in this study. Reduced SAV also served as a key predictor of severe 30-day adverse outcomes. These findings suggest that baseline chest CT could guide timely interventions by aiding in the assessment of disease progression and poor prognoses. Furthermore, patients with visceral obesity or sarcopenia may benefit from regular physical activity and healthy lifestyle habits to reduce the risk of severe infections.

## Supplemental Information

10.7717/peerj.21619/supp-1 Supplemental Information 1 Comparison of body composition between severe and non-severe patients Quantitative variables are presented as the mean ±standard deviation for normally distributed datasets, and as the median (interquartile range) for non-normally distributed datasets.

10.7717/peerj.21619/supp-2 Supplemental Information 2 Comparison of body composition in 65 severe patients with adverse outcomes or not Quantitative variables are presented as the mean ±standard deviation for normally distributed datasets and as the median (interquartile range) for non-normally distributed datasets.

10.7717/peerj.21619/supp-3 Supplemental Information 3 ROC curve predicts severity of viral pneumonia Diagnostic performance of various models for predicting the severity of viral pneumonia. Performance is assessed using the area under the receiver operating characteristic curve (AUC), 95% confidence intervals (CIs), *p*-values, sensitivity, specificity, and the Youden Index.

10.7717/peerj.21619/supp-4 Supplemental Information 4 Calibration curve of the combined predictive model for viral pneumonia severity Predicted probabilities were grouped into deciles, and the observed event rate in each decile was plotted against the corresponding mean predicted probability. The diagonal dashed line represents perfect calibration (y = x). The calibration curve (solid line) showed acceptable agreement between predicted and observed probabilities across most risk quantile intervals, although a slight deviation was observed in the intermediate-risk range.

10.7717/peerj.21619/supp-5 Supplemental Information 5 Internal validation of the combined predictive model using 5 fold cross validation (A–E) Present the receiver operating characteristic (ROC) curves for each of the five folds. The mean area under the curve (AUC) obtained from the 5 fold cross validation was 0.859 (SD = 0.032), suggesting a low risk of overfitting.

10.7717/peerj.21619/supp-6 Supplemental Information 6 Original data

## Abbreviations

CAP: Community-acquired pneumonia
ICU: Intensive care unit
QCT: Quantitative computed tomography
BMI: Body mass index
DICOM: Digital imaging and communications in medicine
CRP: C-reactive protein
T4: 4th thoracic
T8: 8th thoracic
T12: 12th thoracic
Neu%: percentage of neutrophils
Lym%: percentage of lymphocytes
Eos%: percentage of eosinophils
ESV: Erector spinae volume
ESD: Erector spinae density
VAV: Visceral adipose volume
SAV: Subcutaneous adipose volume
-w: body composition measured at the whole vertebral level
-s: standardized body composition (measurements at the whole vertebral level/vertebral slice number)
-I: body composition index (single layer of body composition volume/height^2^)

## Additional Information and Declarations

### Data Availability

The following information was supplied regarding data availability: The raw measurements are available in the Supplemental Files.
