# Supplementary material for: Computed tomography-derived body composition parameters as potential predictors of severity and adverse outcomes in viral pneumonia: a retrospective study
Source: PeerJ. 2026 Aug 11;14:e21619. doi: 10.7717/peerj.21619 (PMC13475389; doi:10.7717/peerj.21619)
Supplement: Supplementary file 1 [file peerj-14-21619-s004.pdf]

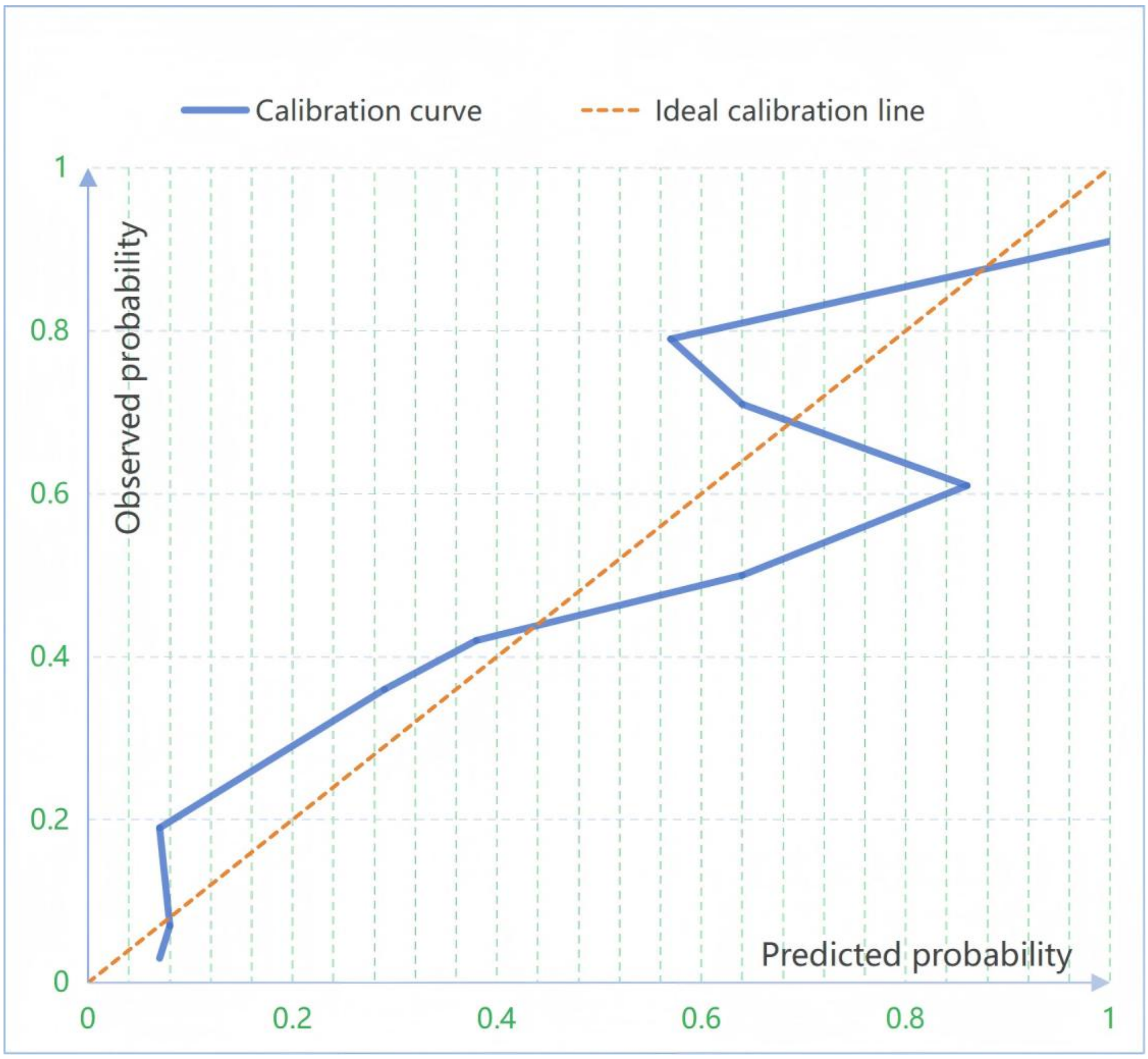
Calibration curve
Ideal calibration line
1
0.8
0.6
0.4
0.2
0
Observed probability
Predicted probability
0
0.2
0.4
0.6
0.8
1
