# Supplementary material for: Computed tomography-derived body composition parameters as potential predictors of severity and adverse outcomes in viral pneumonia: a retrospective study
Source: PeerJ. 2026 Aug 11;14:e21619. doi: 10.7717/peerj.21619 (PMC13475389; doi:10.7717/peerj.21619)
Supplement: Supplementary file 2 [file peerj-14-21619-s005.pdf]

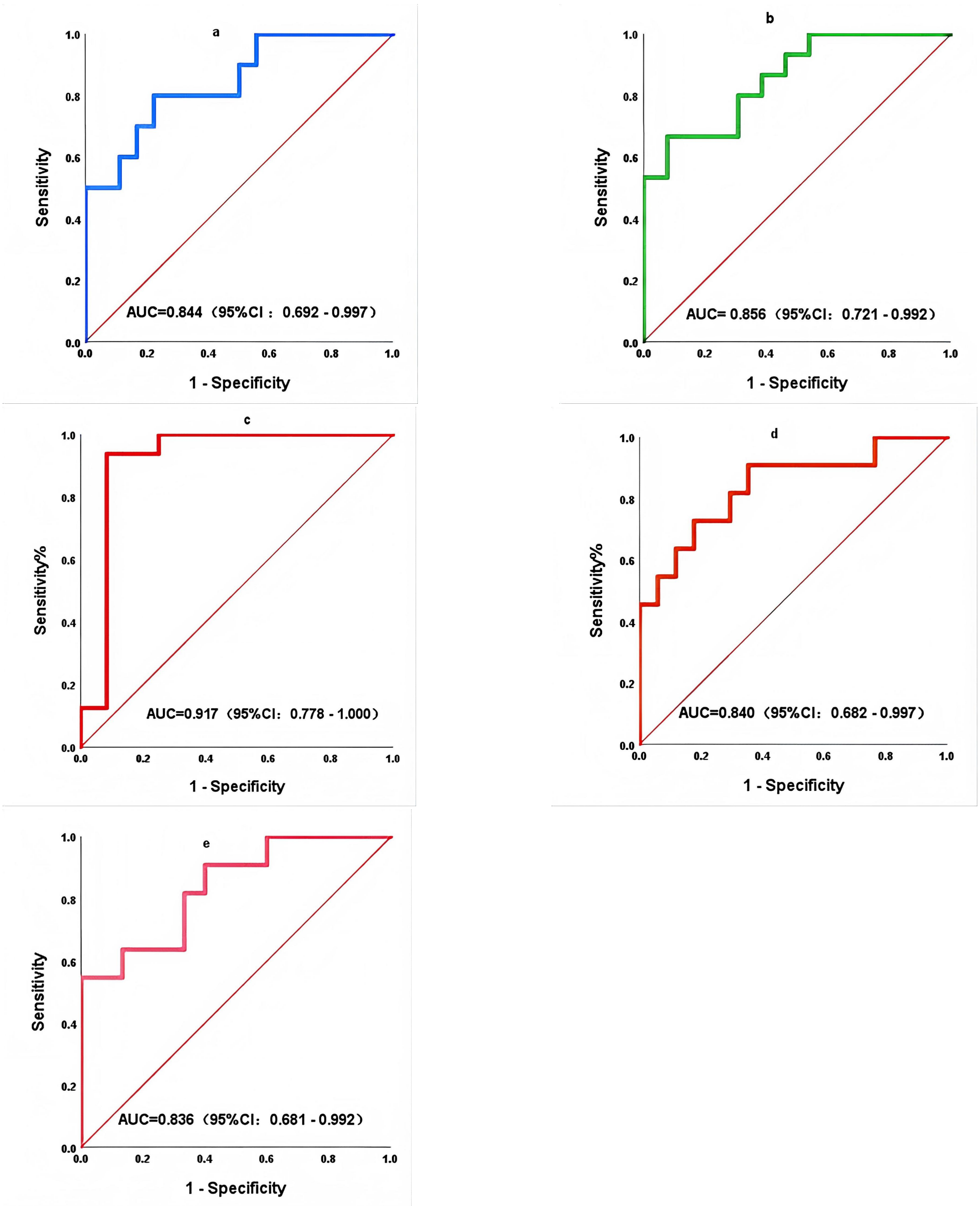
a
Sensitivity
1 - Specificity
AUC=0.844（95%CI：0.692 - 0.997）
b
Sensitivity
1 - Specificity
AUC= 0.856（95%CI：0.721 - 0.992）
c
Sensitivity%
1 - Specificity
AUC=0.917（95%CI：0.778 - 1.000）
d
Sensitivity%
1 - Specificity
AUC=0.840（95%CI：0.682 - 0.997）
e
Sensitivity
1 - Specificity
AUC=0.836（95%CI：0.681 - 0.992）
