# Supplementary material for: Computed tomography-derived body composition parameters as potential predictors of severity and adverse outcomes in viral pneumonia: a retrospective study
Source: PeerJ. 2026 Aug 11;14:e21619. doi: 10.7717/peerj.21619 (PMC13475389; doi:10.7717/peerj.21619)
Supplement: Supplementary file 3 [file peerj-14-21619-s001.docx]

**Supplementary Table 1.** Comparison of body composition between severe and non-severe patients.*(mean±SD or median [IQR])*

|  | All patients (*n*=140) | Non-Severe (*n*=75) | Severe (*n*=65) | *p*-value |
| --- | --- | --- | --- | --- |
| a. Body composition at the costovertebral joints level (Single slice measurement) | | | | |
| T4-TMV | 59.83(47.36–75.98) | 59.79(47.36–74.13) | 59.62(48.52–76.88) | 0.998 |
| T4-TMD | 38.04(31.76–41.06) | 38.08(31.29–40.71) | 37.91(31.78–41.20) | 0.708 |
| T4-PTV | 40.94(33.19–53.49) | 41.93(32.62–53.49) | 39.85(34.06–53.49) | 0.899 |
| T4-PTD | 35.85(29.56–39.89) | 35.45(29.55–39.77) | 36.07(29.45–39.96) | 0.823 |
| T4-PV | 11.26(8.09–15.45) | 11.26(7.95–15.45) | 11.31(8.25–15.47) | 0.965 |
| T4-PD | 30.58(24.38–37.25) | 30.76(23.45–37.09) | 30.65(25.64–37.43) | 0.635 |
| T4-SAV | 42.73(23.90–67.58) | 43.47(23.38–69.83) | 42.73(24.04–59.46) | 0.538 |
| T4-SAD | -88.57(-95.99–-79.14) | -90.11(-96.12–-81.21) | -85.89(-95.99–-78.52) | 0.284 |
| T8-TMV | 26.36(20.49–36.39) | 26.36(20.75–37.20) | 26.88(19.87–36.19) | 0.721 |
| T8-TMD | 27.93±10.14 | 29.54±12.20 | 27.77±10.07 | 0.944 |
| T8-ESV | 5.41(3.97–6.94) | 5.50(4.11–7.00) | 5.40(3.79–6.67) | 0.225 |
| T8-ESD | 22.74(11.96–33.32) | 25.90(11.64–35.02) | 21.82(13.17–28.79) | 0.301 |
| T8-SAV | 42.69(27.99–64.49) | 40.95(25.03–58.74) | 43.20(30.92–67.52) | 0.212 |
| T8-SAD | -89.80(-95.71–-82.44) | -90.20(-95.70–-82.63) | -89.18(-95.91–-80.38) | 0.543 |
| T8-VAV | 7.07(3.72–10.43) | 6.31(3.22–10.10) | 7.30(4.61–11.18) | 0.125 |
| T8-VAD | -76.40(-80.45–-70.16) | -75.92(-80.10–-69.68) | -76.80(-814.61–-70.53) | 0.875 |
| b. Body composition at the whole vertebral levels | | | | |
| T4-TMVw | 269.20(215.30–326.22) | 271.30(215.30–325.01) | 262.76(217.25–346.17) | 0.925 |
| T4-TMDw | 36.94(31.02–40.70) | 37.26(31.02–41.35) | 36.81(30.88–40.46) | 0.922 |
| T4-PTVw | 187.6(152.39–232.11) | 187.9(152.09-232.09) | 187.03(154.25–234.80) | 0.942 |
| T4-PTDw | 35.15(29.23–39.89) | 34.74(28.47–40.43) | 35.37(29.27–39.58) | 0.854 |
| T4-PVw | 54.33(37.83–67.57) | 54.17(37.92–66.27) | 54.15(37.85–68.23) | 0.882 |
| T4-PDw | 30.85(24.64–37.84) | 30.79(23.06–38.38) | 31.05(24.71–37.23) | 0.869 |
| T4-SAVw | 189.9(121.76–301.21) | 198.5(119.65–307.65) | 181.10(122.25–299.34) | 0.662 |
| T4-SADw | -89.50(-96.10–-80.46) | -90.30(-96.00–-82.623) | -85.94(-96.85–-79.75) | 0.363 |
| T8-TMVw | 160.20(123.91–208.18) | 157.30(122.17–203.25) | 160.56(127.15–211.14) | 0.846 |
| T8-TMDw | 27.65±9.87 | 27.66±10.03 | 27.64±9.77 | 0.934 |
| T8-ESVw | 30.65(25.90–40.86) | 31.29(25.44–41.62) | 30.43(25.99–38.56) | 0.381 |
| T8-ESDw | 25.42(16.07–32.92) | 27.11(17.84–34.71) | 23.53(14.49–32.51) | 0.257 |
| T8-SAVw | 252.60(191.73–386.25) | 249.01(160.38–387.01) | 275.10(201.59–386.25) | 0.251 |
| T8-SADw | -89.76(-95.62–-81.58) | -90.58(-95.62–-82.76) | -87.60(-95.95–-80.05) | 0.353 |
| c. Standardized body composition (measurements at the whole vertebral level/vertebral slice number) | | | | |
| T4-TMVs | 67.66(56.04–82.60) | 67.46(54.98–81.40) | 68.37(56.57–84.75) | 0.727 |
| T4-PTVs | 46.71(40.08–58.28) | 46.47(40.08–58.70) | 46.82(39.80–58.28) | 0.909 |
| T4-PVs | 13.64(9.61–16.07) | 13.04(9.69–15.98) | 13.74(9.54–16.51) | 0.690 |
| T4-SAVs | 49.01(30.61–76.09) | 49.84(30.62–76.04) | 46.04(30.56–76.09) | 0.810 |
| T8-TMVs | 32.31(26.36–40.71) | 32.00(27.52–39.24) | 32.11(25.72–41.08) | 0.693 |
| T8-ESVs | 6.27(5.26–8.10) | 6.71(5.51–8.34) | 6.02(5.21–7.56) | 0.071 |
| T8-SAVs | 50.53(39.84–77.61) | 50.20(34.46–79.26) | 54.28(41.08–77.25) | 0.395 |
| T8-VAVs | 9.80±4.71 | 55.68±31.65 | 10.77±4.59 | 0.073 |
| d. Body composition index (measurements at the costovertebral joints level/height^2^) | | | | |
| T4-TMI | 21.93(16.99–27.62) | 21.66(16.82–26.76) | 22.13(17.38–28.23) | 0.998 |
| T4-PTI | 14.66(11.99–18.44) | 14.12(11.68–19.60) | 14.77(12.04–18.12) | 0.889 |
| T4-PI | 4.16(2.95–5.54) | 4.12(2.91–5.49) | 4.21(2.98–5.54) | 0.972 |
| T4-SAI | 15.05(8.93–24.37) | 16.66(8.82–25.35) | 14.39(9.07–21.66) | 0.532 |
| T8-TMI | 9.75(7.39–12.81) | 9.79(7.74–12.91) | 9.89(6.66–12.68) | 0.727 |
| T8-ESI | 1.99±0.73 | 2.07±0.795 | 1.91±0.66 | 0.120 |
| T8-SAI | 15.50(9.87–24.05) | 15.61(8.47–23.44) | 15.39(10.84–24.88) | 0.303 |
| T8-VAI | 2.45(1.40–3.66) | 2.36(1.17–3.44) | 2.66(1.76–3.91) | 0.143 |

**Notes:** Quantitative variables are presented as the mean ± standard deviation for normally distributed datasets, and as the median (interquartile range) for non-normally distributed datasets.

**Abbreviations:** TMV, total muscle volume; TMD, total muscle density; PWV, perithoracic muscle volume; PMD, perithoracic muscle density, PV, pectoral volume; PD, pectoral density; SAV, subcutaneous adipose volume; SAD, subcutaneous adipose density; VAV, visceral adipose volume; VAD, visceral adipose density; ESV, erector spinae volume; ESD, erector spinae density.

-w, body composition measured at the whole vertebral level; -s, standardized body composition (measurements at the whole vertebral level/vertebral slice number); -I, body composition index (single layer of body composition volume/height^2^).
