# Supplementary material for: Computed tomography-derived body composition parameters as potential predictors of severity and adverse outcomes in viral pneumonia: a retrospective study
Source: PeerJ. 2026 Aug 11;14:e21619. doi: 10.7717/peerj.21619 (PMC13475389; doi:10.7717/peerj.21619)
Supplement: Supplementary file 4 [file peerj-14-21619-s002.docx]

**Supplementary Table 2.** Comparison of body composition in 65 severe patients with adverse outcomes or not.*(mean±SD or median [IQR])*

|  | Severe (*n*=65) | Good (*n*=41) | Adverse (*n*=24) | *p*-value |
| --- | --- | --- | --- | --- |
| a. Body composition at the costovertebral joints level (Single slice measurement) | | | | |
| T4-TMV | 62.54±20.75 | 63.36±21.37 | 61.17±20.04 | 0.649 |
| T4-TMD | 37.91(31.78–41.20) | 38.52(32.09–42.15) | 37.06(31.34–40.20) | 0.289 |
| T4-PTV | 39.85(34.06–53.49) | 41.64(34.71–55.05) | 38.58(32.30–51.25) | 0.384 |
| T4-PTD | 36.07(29.45–39.96) | 36.29(29.78–40.48) | 34.63(28.45–39.50) | 0.314 |
| T4-PV | 11.31(8.25–15.47) | 12.58(8.45–15.59) | 10.55(7.03–14.53) | 0.423 |
| T4-PD | 30.65(25.64–37.43) | 29.57(24.13–37.41) | 33.62(25.72–37.52) | 0.714 |
| T8-TMV | 26.88(19.87–36.20) | 25.52(18.27–35.21) | 30.43(21.24–36.42) | 0.392 |
| T8-TMD | 27.77±10.07 | 27.84±10.76 | 27.64±9.01 | 0.885 |
| T8-ESV | 5.36±1.90 | 5.46±1.90 | 5.20±1.92 | 0.603 |
| T8-ESD | 19.96±15.64 | 21.46±14.00 | 17.47±18.10 | 0.303 |
| T8-VAV | 7.30(4.61–11.18) | 7.13(4.92–11.29) | 7.61(3.90–11.18) | 0.683 |
| T8-VAD | -76.80(-814.61–-70.53) | -76.70(-82.60–-68.74) | -76.87(-80.02–-72.02) | 0.892 |
| T12-TMV | 22.12±6.68 | 21.63±6.10 | 22.94±7.62 | 0.520 |
| T12-TMD | 26.65±8.88 | 26.31±9.94 | 27.22±6.94 | 0.639 |
| T12-ESV | 9.18±3.26 | 9.43±2.82 | 8.77±3.91 | 0.414 |
| T12-ESD | 29.26(21.30–34.67) | 29.58(21.11–35.23) | 28.14(21.82–32.71) | 0.734 |
| T12-VAV | 50.39±27.15 | 47.95±21.67 | 54.46±34.57 | 0.870 |
| T12-VAD | -84.58(-89.52–-76.86) | -84.98(-90.96–-80.00) | -84.44(-88.45–-74.59) | 0.271 |
| a. Body composition at the whole vertebral levels | | | | |
| T4-TMVw | 277.26±86.39 | 263.48±88.24 | 300.22±79.77 | 0.099 |
| T4-TMDw | 36.81(30.88–40.46) | 37.21(30.68–41.39) | 36.23(30.88–38.65) | 0.407 |
| T4-PTVw | 195.33±60.15 | 185.65±61.34 | 211.46±55.64 | 0.090 |
| T4-PTDw | 35.37(29.27–39.58) | 35.39(29.04–40.15) | 35.40(29.70–38.03) | 0.719 |
| T4-PVw | 55.95±23.77 | 53.02±24.98 | 60.83±21.22 | 0.199 |
| T4-PDw | 31.05(24.71–37.23) | 29.46(23.17–38.43) | 31.64(26.13–35.83) | 0.765 |
| T8-TMVw | 170.99±60.07 | 165.56±61.37 | 180.04±57.97 | 0.343 |
| T8-TMDw | 27.64±9.77 | 27.47±10.52 | 27.92±8.58 | 0.918 |
| T8-ESVw | 30.43(25.99–38.56) | 29.91(25.30–42.05) | 30.97(26.34–37.63) | 0.724 |
| T8-ESDw | 22.05±14.81 | 23.07±13.54 | 20.36±16.89 | 0.473 |
| T8-VAVw | 51.10(35.65–66.53) | 52.52(37.59–71.76) | 49.78(31.22–65.75) | 0.417 |
| T8-VADw | -76.27(-79.76–-70.19) | -76.94(-81.60–-69.83) | -74.13(-78.02–-70.19) | 0.174 |
| T12-TMVw | 138.23(97.37–201.68) | 131.09(91.72–186.00) | 173.31(101.30–281.70) | 0.089 |
| T12-TMDw | 26.49±9.01 | 26.35±10.31 | 26.72±6.48 |  |
| T12-ESVw | 58.14(44.87–73.19) | 54.89(41.05–71.06) | 61.04(48.49–78.50) | 0.392 |
| T12-ESDw | 27.20(20.80–34.21) | 27.41(20.57–34.20) | 27.10(20.80–34.37) | 0.967 |
| T12-VAVw | 300.30(222.40–452.15) | 261.11(235.71–401.56) | 389.09(158.36–574.56) | 0.341 |
| T12-VADw | -85.27(-90.04–-77.62) | -85.60(-90.66–-79.06) | -83.48(-88.88–-75.70) | 0.254 |
| c. Standardized body composition (measurements at the whole vertebral level/vertebral slice number) | | | | |
| T4-TMVs | 70.27±19.38 | 68.23±19.71 | 73.67±18.71 | 0.276 |
| T4-PTVs | 46.82(39.80–58.28) | 44.89(39.23–57.96) | 48.04(40.96–63.97) | 0.236 |
| T4-PVs | 14.14±5.64 | 13.62±5.80 | 14.99±5.36 | 0.340 |
| T8-TMVs | 33.50±10.09 | 32.87±10.50 | 34.53±9.50 | 0.515 |
| T8-ESVs | 6.02(5.21–7.56) | 5.98(5.26–7.66) | 6.05(4.92–6.90) | 0.828 |
| T8-VAVs | 10.77±4.59 | 11.24±4.59 | 9.99±4.58 | 0.200 |
| T12-TMVs | 30.17±8.66 | 29.55±8.60 | 31.19±8.85 | 0.456 |
| T12-ESVs | 12.02±3.31 | 12.41±3.17 | 11.36±3.50 | 0.237 |
| T12-VAVs | 66.42±30.25 | 65.82±26.26 | 67.40±36.54 | 0.987 |
| d. Body composition index (measurements at the costovertebral joints level/height^2^) | | | | |
| T4-TMI | 22.21±7.02 | 22.52±6.83 | 21.68±7.44 | 0.613 |
| T4-PTI | 14.77(12.04–18.12) | 15.62(12.05–18.59) | 13.63(11.57–17.90) | 0.314 |
| T4-PI | 4.24±1.77 | 4.39±1.76 | 4.00±1.78 | 0.368 |
| T8-TMI | 10.00±3.56 | 9.84±3.62 | 10.27±3.54 | 0.679 |
| T8-ESI | 1.91±0.66 | 1.95±0.67 | 1.82±0.65 | 0.456 |
| T12-TMI | 7.88±2.34 | 7.77±2.13 | 8.08±2.69 | 0.682 |
| T12-ESI | 3.18(2.47–4.13) | 3.39(2.70–4.16) | 2.71(3.09–3.74) | 0.118 |
| T8-VAI | 2.66(1.76–3.91) | 2.59(1.91–3.82) | 2.57(1.46–3.86) | 0.568 |
| T12-VAI | 16.09(10.77–24.92) | 16.09(11.37–24.53) | 17.35(8.81–29.06) | 0.727 |

**Notes:** Quantitative variables are presented as the mean ± standard deviation for normally distributed datasets and as the median (interquartile range) for non-normally distributed datasets.

**Abbreviations:** TMV, total muscle volume; TMD, total muscle density; PWV, perithoracic muscle volume; PMD, perithoracic muscle density; PV, pectoral volume; PD, pectoral density; SAV, subcutaneous adipose volume; SAD, subcutaneous adipose density; VAV, visceral adipose volume; VAD, visceral adipose density; ESV, erector spinae volume; ESD, erector spinae density.

-w, body composition measured at the whole vertebral level; -s, standardized body composition (measurements at the whole vertebral level/vertebral slice number); -I, body composition index (single-layer of body composition volume/height^2^).
