# Supplementary material for: Computed tomography-derived body composition parameters as potential predictors of severity and adverse outcomes in viral pneumonia: a retrospective study
Source: PeerJ. 2026 Aug 11;14:e21619. doi: 10.7717/peerj.21619 (PMC13475389; doi:10.7717/peerj.21619)
Supplement: Supplementary file 5 [file peerj-14-21619-s003.docx]

**Supplementary Table 3.** ROC curve predicts severity of viral pneumonia

| Model name | predictor variable | AUC | 95% *CI* | *p*-value | cut-off value | sensitivity | Specificity | Youden Index |
| --- | --- | --- | --- | --- | --- | --- | --- | --- |
| Clinical model | CRP and Oxygen therapy | 0.796 | 0.723–0.870 | < 0.001 | 0.487 | 0.766 | 0.716 | 0.482 |
| Imaging model 1 | T12-ESV and T12-VAV | 0.735 | 0.651–0.815 | < 0.001 | 0.383 | 0.815 | 0.573 | 0.388 |
| Imaging model 2 | T12-ESDw and T12-VAVs | 0.666 | 0.577–0.756 | < 0.001 | 0.402 | 0.754 | 0.520 | 0.274 |
| Imaging model 3 | T12-ESVs and T12-VAVs | 0.710 | 0.625–0.795 | < 0.001 | 0.429 | 0.754 | 0.600 | 0.354 |
| Imaging model 4 | T12-ESI and T12-VAI | 0.726 | 0.643–0.809 | < 0.001 | 0.385 | 0.815 | 0.573 | 0.388 |
| Combined  model 1 | Clinical model and Imaging model 1 | 0.843 | 0.778–0.909 | < 0.001 | 0.354 | 0.938 | 0.649 | 0.587 |
| Combined  model 2 | Clinical model and Imaging model 2 | 0.834 | 0.767–0.902 | < 0.001 | 0.482 | 0.781 | 0.757 | 0.538 |
| Combined  model 3 | Clinical model and Imaging model 3 | 0.835 | 0.768–0.903 | < 0.001 | 0.395 | 0.875 | 0.676 | 0.551 |
| Combined  model 4 | Clinical model and Imaging model 4 | 0.838 | 0.771–0.905 | < 0.001 | 0.402 | 0.891 | 0.689 | 0.58 |

**Notes:** This table presents the diagnostic performance of various models for predicting the severity of viral pneumonia. Performance is assessed using the area under the receiver operating characteristic curve (AUC), 95% confidence intervals (CIs), p-values, sensitivity, specificity, and the Youden Index.

**Abbreviations:** CRP, C-reactive protein; ESV, erector spinae volume; ESDw, whole-vertebral erector spinae density; VAV, visceral adipose volume; SAV, subcutaneous adipose volume; ESI, erector spinae index; VAI, visceral adipose index.
